# Extraovarian Brenner Tumor in the Vagina: A Case Report and Review of Literature

**DOI:** 10.3390/reports8030103

**Published:** 2025-06-29

**Authors:** Angel Yordanov, Milen Karaivanov, Stoyan Kostov, Vanya Savova, Vasilena Dimitrova

**Affiliations:** 1 Department of Gynaecological Oncology, Medical University Pleven, 5800 Pleven, Bulgaria; drvaniakirilova@abv.bg; 2 Department of General and Clinical Pathology, University Hospital “Dr. Georgi Stranski”, 5800 Pleven, Bulgaria; mkaraivanov1962@gmail.com; 3 Department of Gynaecology, St. Anna University Hospital, Medical University-Varna “Prof. Dr. Paraskev Stoyanov”, 9002 Varna, Bulgaria; drstoqn.kostov@gmail.com; 4 Research Institute, Medical University Pleven, 5800 Pleven, Bulgaria; 5 Faculty of Medicine, Medical University Pleven, 5800 Pleven, Bulgaria

**Keywords:** Brenner tumor, extraovarian, vagina

## Abstract

**Background and Clinical Significance:** Brenner tumors are rare epithelial tumors that can occur in both males and females. They consist of ovarian transition cells surrounded by dense fibrous tissue and can be classified as benign, borderline, or malignant. While most commonly found in the ovary, extraovarian Brenner tumors (EOBTs) have been reported in the uterus, vagina, broad ligament, and omentum. **Case Presentation:** A 71-year-old postmenopausal woman presented with a polypous formation on the upper third of the posterior vaginal wall, which was found at a routine health check. Macroscopically, the lesion appeared as a solid, polypoid mass with a yellowish-gray cut surface, measuring approximately 25 × 20 mm. Histological examination revealed a polypoid formation covered by stratified squamous epithelium, with a dense fibrous stroma (Van Gieson [VG]+) and tubular structures lined by clear epithelial cells. Parenchymal cells showed low proliferative activity, with Ki-67 expression in less than 5% of cells, also Cytokeratin (CK) 7/+/p63:/+/ CK AE1/AE3: /+/ Estrogen Receptor (ER): /+/ and Progesterone Receptor (PR)/−/; CK20/-/; p53/−/, Wilms’ Tumor (WT)-1/−/; Prostate-Specific Acid Phosphatase (PSAP)/−/. The final diagnosis was an extraovarian Brenner tumor. The patient was monitored for two months post-excision, with no signs of recurrence. **Conclusions:** EOBTs are extremely rarely seen and vaginal involvement is far less common. Due to their rarity, these tumors may be confused with other benign or malignant vaginal lesions. In order to differentiate EOBTs from other neoplasms, histological analysis is crucial due to their characteristic transitional-type epithelium and large fibrous stroma. Further studies are required to understand the origin and clinical behavior of EOBTs. Long-term monitoring should be performed to look for any recurrence or malignant change, even though benign Brenner tumors usually have a good prognosis. Awareness of EOBTs and their possible locations is essential for accurate diagnosis and appropriate management.

## 1. Introduction and Clinical Significance

In 1907, Fritz Brenner first described the Brenner tumor [1], arising from granulosa cells in the ovarian follicles. When they develop outside the ovary, they have been referred to as extraovarian Brenner tumors (EOBTs). They are rare epithelial neoplasms that can occur in both males and females. They consist of transitional-type epithelial cells surrounded by dense fibrous stroma and can be benign, borderline, or malignant. In females, few locations of EOBTs have been reported, including the uterus, vagina, broad ligament, and omentum [2]. The exact origin of these tumors remains unclear, with proposed theories suggesting derivation from ovarian celomic epithelium, Walthard nests, mesothelium, and Müllerian or Wolffian remnants [2].

In this study, we report a rare case of an extraovarian Brenner tumor presenting as a polypoid lesion on the posterior vaginal wall in the upper third of the vagina. Pathology and immunohistochemistry were used to analyze the phenotype of its epithelial and stromal components, making the diagnosis.

This case adds to the limited number of reported EOBTs and emphasizes the need for further research into their histogenesis, clinical behavior, diagnostic challenges, treatment, and follow-up.

## 2. Case Presentation

A 71-year-old postmenopausal woman presented with a polypous formation on the upper third of the posterior vaginal wall, discovered during a routine health check. She reported mild vaginal discomfort and discharge. Her medical history was unremarkable, with no significant past illnesses. She had two childbirths, with her last delivery in 1980. Menarche occurred at age 16, with a regular 28-day cycle lasting seven days. Her last menstruation was 15 years ago. A recent Papanicolaou (PAP) test from 2024 showed a PAP II result, indicating no cytological abnormalities.

On physical examination, cardiac and pulmonary assessments were unremarkable, and her vital signs were stable. Abdominal and renal examinations showed no abnormalities. The patient appeared in satisfactory general condition—ECOG 0.

Gynecological examination revealed a 2.5 × 2 cm polyp on the posterior vaginal wall. The uterus was in the anteverted-flexed position, with a normal size and shape, and the adnexal structures were unremarkable. There were no palpable masses or signs of pelvic fluid accumulation. Because of the patient’s symptoms, the decision to excise the tumor’s was made.

### 2.1. Treatment

Under brief intravenous anesthetic, the patient had a vaginal polypectomy. The lesion from the upper third of the posterior vaginal wall was removed by electroloop excision. The patient had a smooth postoperative recovery and was discharged without complications.

### 2.2. Histopathological and Immunohistochemical Findings

#### 2.2.1. Macroscopic Description

The excised specimen consisted of a polypous formation measuring 25 × 20 mm.

#### 2.2.2. Microscopic Description

**Figure 1:**
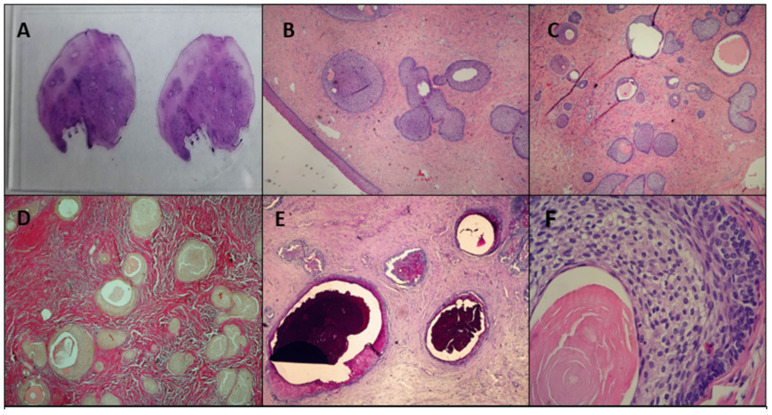
Pathological findings of the vaginal EOBT: (**A**) macroscopic view of the histological specimen H&E; (**B**) microscopic ×10 H&E; (**C**) microscopic ×10 H&E; (**D**) microscopic ×10 Van Gieson; (**E**) microscopic ×10 PAS—Alcyan blue; (**F**) microscopic ×100 H&E.

Histological examination revealed a polypous lesion covered by stratified squamous epithelium. The underlying stroma was dense and fibrous (VG+), consistent with a well-developed connective tissue component. The tumor contained tubular structures lined with clear epithelium, resembling urothelium or squamous epithelium, with a palisading arrangement at the periphery. Some gland-like structures contained PAS-positive secretions, further supporting the characteristic features of a Brenner tumor (Figure 1E). Figure 1 presents the macroscopic view of the histological specimen (Figure 1A) and the microscopic appearance, including magnification and staining of the specimen (Figure 1B–F).

#### 2.2.3. Immunohistochemistry

**Figure 2:**
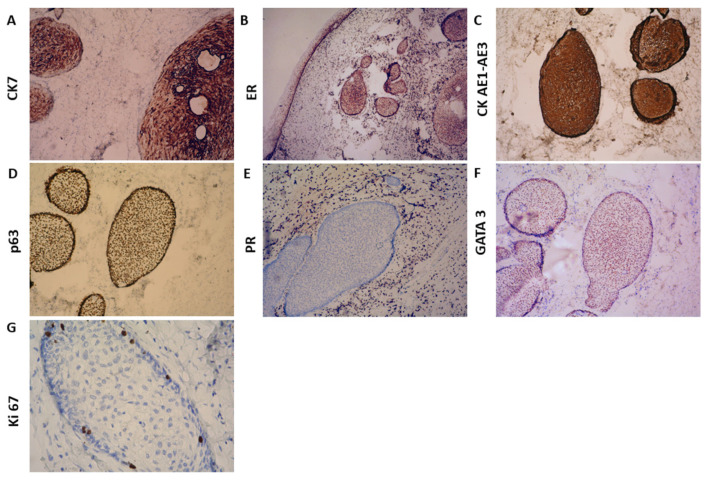
Immunohistochemical findings: (**A**) ×25 CK7; (**B**) ×ER; (**C**) ×25 CK AE1-AE3; (**D**) ×25 p63; (**E**) ×25 PR; (**F**) ×25 GATA 3; (**G**) ×25 Ki 67.

Immunohistochemical analysis revealed positive staining for Cytokeratin (CK) 7 (Figure 2A), p63 (Figure 2D), CK AE1/AE3 (Figure 2C), Estrogen Receptor (ER) (Figure 2B), and GATA3 (moderate to weak staining in the stroma) (Figure 2F). In contrast, Progesterone Receptor (PR) (Figure 2E), CK20, p53, WT-1, and Prostate-Specific Acid Phosphatase (PSAP) were negative. Ki-67 staining was positive in less than 5% of epithelial and stromal cells, indicating a low proliferative index (Figure 2G).

#### 2.2.4. Follow-Up

After surgery, the patient recovered well and showed no recurrence after 2 months of follow-up.

## 3. Discussion

**Figure 3:**
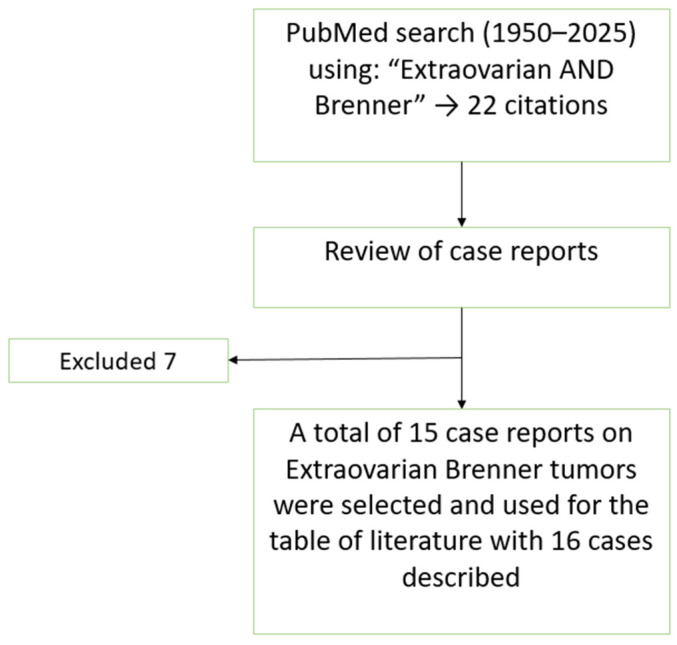
Flowchart of the literature search and selection process for the case reports included in the review and discussion.

Extraovarian Brenner tumors (EOBTs) are exceptionally rare, with only a limited number of cases reported in the literature. To identify previously reported cases of EOBTs, we performed a literature search using PubMed and Google Scholar with the terms ‘extraovarian Brenner tumor’, ‘vaginal Brenner tumor’, and ‘extraovarian transitional cell tumor’. Reports in English from 1950 to 2024 were included. Sixteen cases met these criteria and are summarized in Table 1. A flowchart illustrating the literature search strategy and selection process is presented in Figure 3. The table consists of 16 cases of EOBTs in females, including the present case, focusing on patient demographics, tumor location and size, IHC, symptoms, treatment, and follow-up outcomes.

The following conclusions are observed:

The patients’ range in age from 30 to 84 years, with an average of 61.1 years. The majority of cases occur in postmenopausal women, suggesting a higher prevalence in older individuals. Half of the cases are in the 51–65-year-old range. Only two cases were described in women under 50. EOBTs predominantly affect postmenopausal women, with most cases occurring in the 51–65 age range.

The most common tumor location appears to be the vagina, with lesions reported in the upper, middle, or lower thirds of the vagina. In most instances, the tumors are described as polypoid masses or incidental findings during pelvic exams, reflecting a lack of clinical symptoms. Broad ligament involvement is also frequent. The uterus accounted for only three cases, and one case was described in the omentum.

A total of 10 cases, including size data, were reported. The smallest tumor was 0.5 cm, the largest—9 cm, and the average tumor size (for reported cases) was 2.9 cm. Most EOBTs are small (under 3 cm), but some can be significantly larger (up to 9 cm). The largest tumor (9 cm) was found in the omentum.

The majority of cases are incidental discoveries. The majority of tumors are asymptomatic and discovered incidentally, but some present with chronic vaginal symptoms. Bleeding, irritation, and soreness are the primary symptoms when present. Only one case was linked to procidentia (pelvic organ prolapse) and only one case represents the instance of a long-standing vaginal mass without symptoms. This suggests that many of these tumors are asymptomatic and are often found during routine examinations or surgeries.

Immunohistochemical analysis of this case revealed a profile that aligns with the limited data available from prior reports. Positive staining for CK7, p63, CK AE1/AE3, ER, and GATA3, combined with low Ki-67 proliferation (<5%), supports the diagnosis of a benign extraovarian Brenner tumor with transitional-type epithelium. Importantly, negative staining for PR, CK20, WT-1, p53, and PSAP helps exclude other tumor variants. In comparison to the few cases reporting detailed IHC profiles in the literature, our findings correlate with the most frequent markers observed (GATA3, p63, and CK7), which reflect urothelial differentiation. The absence of PAX8 expression, commonly used to mark the Müllerian epithelium, supports the extra-Müllerian differentiation of this tumor.

Most cases had no documented follow-up. Four cases with follow-up showed no recurrence within 2–18 months. The available data suggest a low recurrence rate, but follow-up is lacking in most cases. 

This analysis of 16 cases of EOBTs provides insights into their epidemiology, presentation, management, and follow-up. The average age is 61.1 years, with most cases occurring in postmenopausal women. The vagina and broad ligament are the most common sites. Most tumors are small (1–5 cm) and asymptomatic, identified as an incidental finding. Hysterectomy is used for most of the uterine cases, while excision is preferred for vaginal/broad ligament cases. A good prognosis is indicated by low recurrence rates, but in most cases, follow-up data are not available. 

The present case (a 71-year-old patient with a 2.5 × 2 cm vaginal tumor, discovered incidentally and treated with excision, with no recurrence after two months) matches with common cases. The patient’s age is near the reported average of 61.1 years. The tumor’s location in the vagina matches the most frequent site, and its size (2.5 × 2 cm, approximately 2.3 cm average) is close to the mean size of 2.9 cm. Incidental findings, as in this case, are the most common detection method. The treatment method performed was excision, the most common treatment method. Finally, the two-month follow-up without recurrence is comparable to the outcomes observed in other excised cases.

There is also one described case of Extraovarian malignant Brenner Tumor, but it is not included in the table and overview due to a lack of data [16].

## 4. Conclusions

Extremely uncommon, extraovarian Brenner tumors (EOBTs) are exclusive to the vagina. This case report expands to the literature of EOBTs by describing a 71-year-old postmenopausal woman with an accidentally discovered vaginal polypoid mass that turned out to be a benign Brenner tumor. A review of previous EOBT indicates that these tumors are usually asymptomatic, mostly impact postmenopausal women, and are usually discovered by coincidence during routine checks. The majority of reported tumors are small (less than 3 cm), and the main treatment is surgical removal. The immunohistochemical profile presented here expands the current understanding of EOBTs and offers practical diagnostic markers to aid in distinguishing them from neoplasms with similar morphologies. Long-term surveillance is recommended to rule out any malignant transformation or recurrence, even if the available follow-up data indicates a low recurrence rate.

This case highlights the need for additional research to fully comprehend the pathophysiology, clinical behavior, and best management practices of EOBTs, as well as the need to take them into account when making a differential diagnosis of vaginal masses.

## Figures and Tables

**Table 1:** EOBTs in females.

№	Age	Location	Tumor Size	Finding	Treatment	IHC (+)	IHC (−)	Follow-Up
Robinson et al. [3]	64	Left broad ligament	n.a.	Incidental	Total hysterectomy	n.a.	n.a.	n.a.
Arhelger et al. [4]	55	Uterine corpus	1.3 cm	Incidental	Vaginal hysterectomy	n.a.	n.a.	n.a.
Wagner et al. [5]	53	Left broad ligament	n.a.	Incidental	Total hysterectomy	n.a.	n.a.	n.a.
Chen et al. [6]	67	Vagina, mid third	n.a.	Incidental	n.a.	n.a.	n.a.	n.a.
Pschera et al. [7]	30	Right broad ligament	n.a.	Incidental	n.a.	n.a.	n.a.	n.a.
Hampton et al. [8]	64	Right broad ligament	n.a.	Procidentia	n.a.	n.a.	n.a.	n.a.
Rashid et al. [9]	77	Vagina, not specified	2 × 1.8 cm	Irritation and soreness	Excision	n.a.	n.a.	18 months-no recurrence
Ben-Izhak et al. [10]	68	Vagina, upper third	0.5 cm	Incidental	Hysterectomy	n.a.	n.a.	n.a.
Ben-Izhak et al. [10]	72	Vagina, mid third	1.2 cm	Vaginal bleeding	n.a.	n.a.	n.a.	n.a.
Angeles-Angeles et al. [11]	63	Uterus cavity	n.a.	Postmenopausal uterus bleeding	n.a.	n.a.	n.a.	n.a.
Shaco-Levy et al. [12]	84	Vagina, lower third	1.8 cm	Vulvar irritation and soreness	n.a.	n.a.	n.a.	n.a.
Hwang et al. [13]	43	Omentum	9 cm	Incidental	Excision	CK-PAN;	CD34; CD10, CK20; calretinin, c-KIT; DOG-1; PAX-8	17 months-no recurrence
						P63; WT-1;		
						CK7; GATA-3;		
						UPK3		
Park et al. [14]	76	Vagina, not specified	2.5 cm	Incidental	n.a.	GATA-3;	PAX8	n.a.
						p63; ER		
Rui-Yue Hu et al. [2]	53	Uterine corpus	5.8 × 5.7 × 4.6 cm	Incidental	Excision	CK5/6; GATA-3 34βE12; P63;	PAX8; p53;	2 months-no recurrence
						ER; PR;	p16	
						cyclinD1		
Zhang et al. [15]	64	Vagina, lower third	1.5 × 1.5 × 0.5 cm	History of 9 years vaginal mass, no complain	Excision	GATA-3;	CK-20; PSA; p16; PAX-8	3 months-no recurrence
						P63; ER; PR; Vimentin		
Present case	71	Vagina, upper third	2.5 × 2 cm	Incidental	Excision	Ki-67; CK 7; p63; CK AE1/AE3; ER; GATA3	PR; CK20; p53, WT-1; PSAP	2 months-no recurrence

## Data Availability

Data presented in this study are available on request from the corresponding author due to privacy concerns.

## References

[1] Brenner F. (1907). Ds oophoroma folliculare. Frankf. Z. Pathol..

[2] Hu R.-Y., Deng Y.-J., Zhu H.-H., Zhou J., Hu M., Liang X.-Q., Xiao Q.-J., Zhou L., Peng X.-Y., Zhang X.-W. (2020). Extraovarian Brenner tumor in the uterus: A case report and review of literature. Diagn. Pathol..

[3] Robinson T.G. (1950). Extra-ovarian brenner tumour. J. Obstet. Gynaecol. Br. Emp..

[4] Arhelger R.B., Bocian J.J. (1976). Brenner tumor of the uterus. Cancer.

[5] Wagner I., Bettendorf U. (1980). Extraovarian brenner tumor. Case report and review. Arch. Gynecol. Obstet..

[6] Chen K.T. (1981). Brenner tumor of the vagina. Diagn. Gynecol. Obstet..

[7] Pschera H., Wikström B. (1991). Extraovarian Brenner Tumor Coexisting with Serous Cystadenoma. Case report. Gynecol. Obstet. Investig..

[8] Hampton H.L., Huffman H.T., Meeks G.R. (1992). Extraovarian Brenner tumor. Obstet Gynecol..

[9] Rashid A.M., Fox H. (1995). Brenner tumour of the vagina. J. Clin. Pathol..

[10] Ben-Izhak O., Munichor M., Malkin L., Kerner H. (1998). Brenner Tumor of the Vagina. Int. J. Gynecol. Pathol..

[11] Angeles-Angeles A., Gutiérrez-Villalobos L.I., Lome-Maldonado C., Jiménez-Moreno A. (2002). Polypoid Brenner Tumor of the Uterus. Int. J. Gynecol. Pathol..

[12] Shaco-Levy R., Benharroch D. (2013). Vaginal Brenner Tumor. Int. J. Gynecol. Pathol..

[13] Hwang C.S., Lee C.H., Lee S.J., Kim Y.G., Kim A., Park D.Y., Kang H.J., Shin D.H. (2017). A peculiar case report of extraovarian Brenner tumor arising in the omentum. World J. Surg. Oncol..

[14] Park S., Cho M.S. (2017). Vaginal Brenner tumor with literature review: Does this tumour originate from Walthard nests?. Malays. J. Pathol..

[15] Zhang Q., Tian C., Wang K., Xin Q., Shen Y., Zhang C.-S., Ma Z. (2020). A case of a vaginal Brenner tumor without a gland mimicking a borderline tumor: Unusual morphology and diagnostic pitfalls. J. Int. Med. Res..

[16] Chekanov M.N., Kuz’min I.V., Bulycheva I.V., Nobles E., Izupova N., Rutkovskiĭ E.A., Iakushenko V.K. (2004). Ekstraovarial’naia zlo-kachestvennaia opukhol’ Brennera [Extraovarian malignant Brenner tumor]. Arkhiv Patol..

